# Next steps in studying host-microbiome interactions in apical periodontitis

**DOI:** 10.3389/froh.2023.1309170

**Published:** 2023-12-01

**Authors:** Athina C. Georgiou, Bernd W. Brandt, Suzette V. van der Waal

**Affiliations:** ^1^ Department of Endodontology, Academic Centre for Dentistry Amsterdam (ACTA), University of Amsterdam and Vrije Universiteit Amsterdam, Amsterdam, Netherlands; ^2^ Department of Preventive Dentistry, Academic Centre for Dentistry Amsterdam (ACTA), University of Amsterdam and Vrije Universiteit Amsterdam, Amsterdam, Netherlands

**Keywords:** apical periodontitis, microbiota, inflammation, public health, endodontic infection

Apical periodontitis (AP) is an inflammatory response around the root tip of a tooth after invasion of microorganisms into the dental-pulp space, which has led to pulp necrosis and infection of the entire root-canal space (1). Invasion of microorganisms into a tooth can occur as a result of loss of integrity of the tooth after caries or trauma. Different species of microorganisms present themselves at the root apex and, as a result, inflammation occurs that continues perpetually around the root apex. AP can occur with symptoms of pain, swelling and/or loss of function, or it is asymptomatic and is then discovered on radiographic examination of the oral cavity. Without intervention, such as tooth extraction or root-canal treatment, this inflammatory response can last for years and can also result in different types of inflammation (2). When treatment does not take place or fails to succeed, the body is constantly producing an inflammatory response to the bacteria presented at the apex, in order to contain the infection and to maintain homeostasis. According to a study published in 2020, the prevalence of AP has increased during the last years in the adult general population compared with data from 2012 (6.3% vs. 5.4%) in both endodontically treated (41.3% vs. 35.9%) and untreated teeth (3.5% vs. 2.1%) compared to previous studies performed. This indicates how common AP is in the general population (3) and also the potential unknown effect that this could have to systemic disease promotion.

Without intervention, this continuous inflammatory response and the presence of microorganisms may also cause systemic effects. There is emerging evidence that the consequences of oral pathosis, like marginal or apical periodontitis, are not limited to discomfort or pain, but also to the increase or suppression of some inflammatory markers in peripheral blood (4, 5). The mechanisms that link an oral infection to systemic consequences involve bacteriaemia (i.e., the spread of bacterial byproducts in the blood stream), and enhanced low-grade inflammation. Recently, studies on the systemic inflammation in healthy individuals measuring a range of inflammatory mediators have been undertaken (6, 7). These studies conclude that AP contributes to changes in the levels of molecular markers of inflammation. It has also been shown that a dose-response relationship exists between AP and the immunologic markers in blood: the bigger the lesion or the larger the number of lesions, the larger the inflammatory response is (4). In marginal periodontitis, the surface area of inflammation is larger than the rather localized lesions in AP (8). This is a limitation in the exploration of the causality of systemic conditions as a result of AP. Nevertheless, these recent studies do report differences in inflammatory markers between subjects with or without AP. Given the ageing of the population and the high prevalence of AP, it is essential and a matter of prevention in public health to seek some kind of treatment, even when the AP is asymptomatic.

In systemically healthy individuals, the ongoing inflammatory response to the endodontic infection is aimed to maintain homeostasis. Homeostasis is a (inflammatory) response of the body to external stimuli and it aims to maintain internal stability or balance (9). However, what happens when homeostasis comes under pressure and balance seems to be lost? How does the body then cope with the external problem, that is the endodontic infection? Often patients experience pain at the site of the AP lesion when or after they have been ill (e.g., influenza or bacterial infection), or when their health is compromised by stress or fatigue. There is evidence that the presence or absence of pain at different moments relates to a different microbial composition at the AP site (10, 11). Hence, the question that arises is: do the microorganisms differ due to the inflammatory response or does the inflammatory response differ due to a different microbial composition?

Bidirectional interactions exist: the microorganisms affect the host and the host affects the microorganisms. Recent research has investigated the effect of comorbidities, such as rheumatoid arthritis or cardiovascular diseases, with AP and report an association of elevated levels of inflammatory markers: analyses of the levels of cytokine/chemokine markers in AP lesions indicated that these markers may be associated with the differentiation of different T-cell populations (12). Cytokines or chemokines are signaling proteins that control the inflammatory response. In addition to the comorbidities, the immune system is also affected by age, and seasonal changes. Therefore, the fitness or resilience of the immune system has an effect on the inflammatory response at the root tip (13). Considering that these proteins are also changing the nutrients available at the root tip, they also affect the microbiota of the root-canal system. How this, or different comorbidities, changes the microbiota is a subject for future research.

The composition of the endodontic microbiota is the result of a compositional shift of oral communities, after caries or trauma (14). During the first phases of the infection, oxygen and an abundance of nutrients are available from the oral cavity that allow facultative bacteria to grow. Later, as the endodontic infection matures, the lack of sugar and oxygen, and the availability of proteins and amino acids (e.g., pulp remnants, inflammatory response of the host, dead microorganisms) allow other types of bacteria to flourish. The microbial succession and the changes to ecological conditions is a dynamic process that depends on the local availability of nutrients and the initial inoculum of the infection, deriving from the oral microbiota of the host (14). Microorganisms establish and colonize the entire root-canal system and are organized in biofilms that make them less susceptible to treatments. In addition, besides on the inside of the root canal, biofilms have also been found on the external surface of root tips, the extra-radicular infection (15). The extra-radicular infections are one of the reasons of failure of the conventional root-canal treatments and have also specific ecological conditions. Therefore, it is understandable that an intervention, such as a root-canal treatment, changes the ecological conditions (16), resulting into a different microbial composition of the persistent biofilm after a failed treatment (17).

An inflammatory response is caused not only by the microorganisms themselves, but also by their byproducts. Hence, it is essential to understand these byproducts and their functions using metabolomic/metaproteomic in addition to microbial evaluations. Already proteins involved with resistance to antibiotics, proteases, adhesins and exotoxins have been identified (18–20). How the byproducts influence the immune system and may cause a systemic problem is by far not yet fully explored. In periodontology, there are some early new insights into the causality of Alzheimer's disease and oral bacteria, namely *Porphyromonas gingivalis*, and their byproducts (21). *Porphyromonas gingivalis*, a very well-known pathogen in chronic periodontitis, has been identified in the brain of Alzheimer's disease patients (21). In the same cohort, toxic proteases from this bacterium called gingipains were also identified in the brain of Alzheimer's patients (21). Considering that AP and periodontitis have a similar microbial and immunologic profile, it is highly possible that AP also contributes in a similar way to systemic conditions. This hypothesis is yet to be explored.

The last two decades, with the development of high-throughput DNA-sequencing technologies, several studies analyzed the microbial composition of root-canal infections, especially utilizing 16S-rRNA gene sequencing (22). The sampling methods differed in the different studies, from paper points and endodontic files to extraction and pulverization of the whole tooth. Sampling of the root-canal system with files or paper points does not represent the real situation as for its inability to reach the whole biofilm in for instance the apical portion and its ramifications, thereby somewhat limiting the sampling to accessible parts of the root-canal system and planktonic bacteria from the main root canal (22). However, paper-point sampling is a quick and easy method and could be useful to study the microbiota when developing *in vitro* models and testing antimicrobial strategies. To study the effect of AP on systemic health, it is essential to study the microbiota of the root apex especially. However, the apical part can be the most complexly shaped part of the root-canal system. Therefore, we consider cutting the root tip and cryo-pulverization of the apical part as the most relevant method, since then the entire root-canal infection is retrieved.

A further step should be taken to understand the functional capacity and functions of these microorganisms by performing metagenome shotgun and metatranscriptome sequencing. The first study employing such a method in endodontology was published recently giving us information on the microbial functions of the bacteria in the root-canal, utilizing paper points as a sample method (23). Metagenome shotgun analysis, metatranscriptomics and proteomics are the next steps in unravelling the interactions between the bacteria and their (by)products and the immune system. Larger sample sizes, including AP cases in different stadiums (e.g., initial problem, established AP lesions, symptomatic, asymptomatic), of people of various ages, and of different genetic background, are needed in a stratified design. Another factor that could help us to understand the role of the AP microbiota, is to simultaneously sample the apex of the AP tooth and the blood of the individuals, in order to study the translocation of the bacteria involved to peripheral blood (24). Information from all these sources can then be used to model the disease and to study how the system recovers after therapy.

Understanding the microbiota of AP, and the subsequent inflammatory response, may result in a model which allows us to study host-microbiome interactions. Since AP, in contrast to marginal periodontitis, can completely be resolved, we believe that AP can be a promising model to not only study the effect of oral disease on systemic health, but also to study how the immune system reacts and adapts to various challenges at the different stages of life. These inflammation model studies can be carried out as clinical trials with human subjects, when AP is treated, but possibly also in reverse in animal studies, in which apical periodontitis is induced. In this way, we could study the onset and repair mechanisms that are involved. No matter which model is chosen, for the sake of public welfare and prevention of disease, the interaction between host, oral infections and their microbiome is an important subject for future investigation.
